# UPLC/Q-TOF MS-Based Metabolomics and qRT-PCR in Enzyme Gene Screening with Key Role in Triterpenoid Saponin Biosynthesis of *Polygala tenuifolia*

**DOI:** 10.1371/journal.pone.0105765

**Published:** 2014-08-22

**Authors:** Fusheng Zhang, Xiaowei Li, Zhenyu Li, Xiaoshuang Xu, Bing Peng, Xuemei Qin, Guanhua Du

**Affiliations:** 1 Modern Research Center for Traditional Chinese Medicine Shanxi University, Taiyuan, Shanxi, China; 2 College of Chemistry and Chemical Engineering, Shanxi University, Taiyuan, Shanxi, China; 3 Beijing Institute of Traditional Chinese Medicine, Beijing Hospital of Traditional Chinese Medicine Affiliated to Capital Medical University, Beijing, China; 4 Institute of Materia Medica, Chinese Academy of Medical Sciences, Beijing, China; Università della Calabria, Italy

## Abstract

**Background:**

The dried root of *Polygala tenuifolia*, named Radix Polygalae, is a well-known traditional Chinese medicine. Triterpenoid saponins are some of the most important components of Radix Polygalae extracts and are widely studied because of their valuable pharmacological properties. However, the relationship between gene expression and triterpenoid saponin biosynthesis in *P. tenuifolia* is unclear.

**Methodology/Findings:**

In this study, ultra-performance liquid chromatography (UPLC) coupled with quadrupole time-of-flight mass spectrometry (Q-TOF MS)-based metabolomic analysis was performed to identify and quantify the different chemical constituents of the roots, stems, leaves, and seeds of *P. tenuifolia*. A total of 22 marker compounds (VIP>1) were explored, and significant differences in all 7 triterpenoid saponins among the different tissues were found. We also observed an efficient reference gene GAPDH for different tissues in this plant and determined the expression level of some genes in the triterpenoid saponin biosynthetic pathway. Results showed that MVA pathway has more important functions in the triterpenoid saponin biosynthesis of *P. tenuifolia*. The expression levels of squalene synthase (SQS), squalene monooxygenase (SQE), and beta-amyrin synthase (β-AS) were highly correlated with the peak area intensity of triterpenoid saponins compared with data from UPLC/Q-TOF MS-based metabolomic analysis.

**Conclusions/Significance:**

This finding suggested that a combination of UPLC/Q-TOF MS-based metabolomics and gene expression analysis can effectively elucidate the mechanism of triterpenoid saponin biosynthesis and can provide useful information on gene discovery. These findings can serve as a reference for using the overexpression of genes encoding for SQS, SQE, and/or β-AS to increase the triterpenoid saponin production of *P. tenuifolia*.

## Introduction

Radix Polygalae, which is the root of *Polygala tenuifolia* Willd. or *P. sibirica* L. (Fam. Polygalaceae) as listed in Chinese Pharmacopoeia (2010 edition), has been used in traditional medicine for tonic, sedative, antipsychotic, and expectorant purposes for centuries in China [1], [2]. The studies on the chemical components of this herb have mainly focused on various saponins, xanthones, and oligosaccharide esters [3]–[6]. Among them, triterpenoid saponins are documented as an important group of active components. The structures of the triterpenoid saponins in *P. tenuifolia* are rather complex [7]. The triterpenoid saponins have a triterpene presenegenin aglycone as basic structure, and the slight differences among these saponins are a result of various substitutes including saccharides and/or acyl groups linked to C-28. Considering the complicated isolation and complete structural characterization of triterpenoid saponins, more methods need to be established to analyze the chemical constituents of triterpenoid saponins in *P. tenuifolia*. In recent years, UPLC coupled with electrospray ionization quadrupole time-of-flight mass spectrometry (Q-TOF MS) has been widely used as a powerful tool in the analysis of complex saponin metabolites because this tool shortens the analysis time and provides accurate mass measurements [8]. Our report, which is an investigation of constituents in *P. tenuifolia* by ultra-performance liquid chromatography (UPLC) /Q-TOF MS, aims to analyze the peak area intensity differences of triterpenoid saponins among roots, stems, leaves, and seeds, and quantify their metabolites using metabolomic technology.

Modern pharmacological studies have already demonstrated that these triterpenoid saponins exhibited a wide variety of activities, such as cognitive improving, anti-angiogenic, hypolipidemic, anti-inflammatory, anti-depressive, anxiolytic, sedative-hypnotic, and neuroprotective actions [2], [9]–[16]. Although various chemical and pharmacological properties of triterpenoid saponins have been extensively studied, there is no study that has focused on the biosynthetic pathway of the triterpenoid saponins in Radix Polygalae. The method of terpenoid biosynthesis in plants is through the isoprenoid pathway by cyclization of 2, 3-oxidosqualene to primarily give cucurbitane skeleton. Two biosynthetic pathways are possible for presenegenin skeleton: the mevalonate (MVA) in cytosolic pathway and the non-mevalonate or 2-C-methyl-D-erythritol 4-phosphate/1-deoxy-D-xylulose 5-phosphate (MEP/DOXP) in plastidial pathway [17]. The MEP pathway is absent in higher animals and fungi, but in green plants, the MEP and MVA pathways co-exist in separate cellular compartments [18]. The triterpenoid saponin skeleton may undergo various oxidations, substitution, and glycosylation mediated by cytochrome P450-dependent monooxygenases (CYP 450s), glycosyltransferases (UGTs), and other enzymes [19]. To date, only a few publications that report the identification of CYP 450s and UGTs involved in biosynthesis of triterpenoid saponins are available. Characterized CYP450s and UGTs, such as CYP 93E1, CYP 88D6, CYP 51H10, CYP 72A154, CYP 716A12, UGT 73P2, UGT 74M1, UGT 73K1, and UGT 73F3, have been reported to be involved in modification of triterpenoid saponins [20]. In nucleotide database of NCBI from *P. tenuifolia* (up to 2014.02), only three unigenes and two complete cDNAs had been identified and whether enzymes have an important function in the biosynthesis pathway of triterpenoid saponins is still unknown. If we continue to figure out the whole biosynthesis pathway of triterpenoid saponins, many enzyme genes in triterpenoid saponin biosynthesis are needed to be screened by the gene expression of these enzymes for further analysis.

Gene expression analysis has been frequently used to analyze mRNA in different organisms, tissues, and in several transgenic and gene mutation experiments [21]–[24]. Individual RNAs can be measured by Northern blot [25], RNase protection analysis [26], and in situ hybridization [27]. However, these techniques are time-consuming and/or require large quantities of RNA for detection [28]. Quantitative real-time polymerase chain reaction (qRT-PCR) has a broad range of detection with advantages in repeatability, sensitivity, and specificity. It is also valuable and easy to use when studying rare transcripts and detecting low abundance mRNAs [29]. Given the substantial variations in RNA's stability, quantity, and purity, and the efficiency of reverse transcription and PCR, the reliability of qRT-PCR results is highly dependent on the reference genes chosen. However, many of these reference genes may not be stably expressed under all conditions [30]–[32]. Some reference genes have been reliably validated in one plant species but not well suited in others [33]. Therefore, experimental proof is suggested when reference genes are to be used under a new experimental condition or in a new plant species [34]. Moreover, given the limitations of Sanger sequencing [36]–[37], expressed sequence tag (EST) analysis has identified only five mRNAs, which are all involved in biosynthesis of the saponins. The next-generation sequencing technology, known as RNA-seq, emerged as an effective approach for high-throughput sequence, now facilitates studies of transcriptome in a rapid way and has been used to explore gene structure and expression profiling on traditional Chinese medicine [38]–[39]. In our previous research, the transcriptome of Radix Polygalae was evaluated by Illumina HiSeq 2000 (data not shown), which provides a basis for further research about gene expression analysis of *P. Tenuifolia*.

In the present study, the differences among chemical components of triterpenoid saponins in different tissues from the roots, stems, leaves, and seeds of *P. tenuifolia* were identified by UPLC/Q-TOF MS. qRT-PCR was further performed to screen reference genes (Table S1) and measure the expression of nine relevant genes (Table S2) in triterpenoid saponin backbone biosynthesis pathway (from acetyl-CoA or pyruvate to sapogenin) of *P. tenuifolia*. The expression levels of three CYP 450s and three UGTs genes (Table S3) were also analyzed. Those six genes were all from the CYP 450s and UGTs families which were identified to be involved in biosynthesis of triterpenoid saponins and were also found in the transcriptome of Radix Polygalae. This study aimed to discover the differential gene expression in different tissues, and explore the excellent genetic traits in the triterpenoid saponin biosynthesis pathway. The results may provide useful information for deeper understanding of the biosynthesis pathway of triterpenoid saponins in *P.* t*enuifolia*.

## Results

### Metabolite analysis of *P. tenuifolia* by UPLC/Q-TOF MS

**Figure 1:**
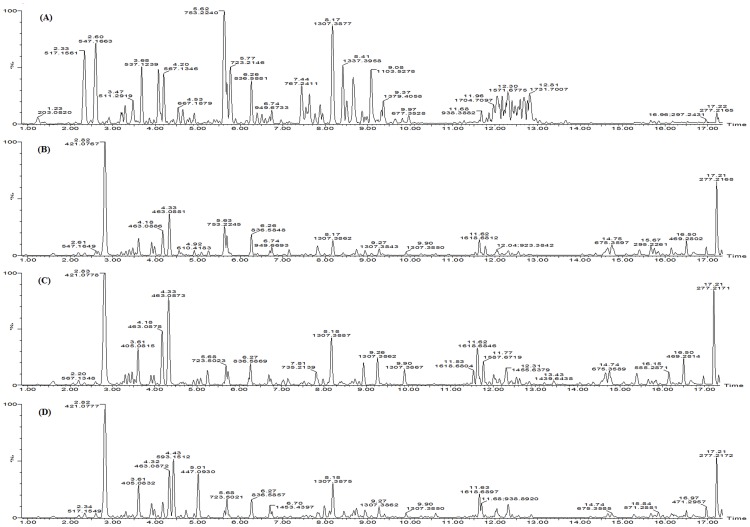
MS TIC chromatograms of roots (A), stems (B), leaves (C), and seeds (D) of *P. tenuifolia* by UPLC/Q-TOF MS in negative ion mode.

**Figure 2:**
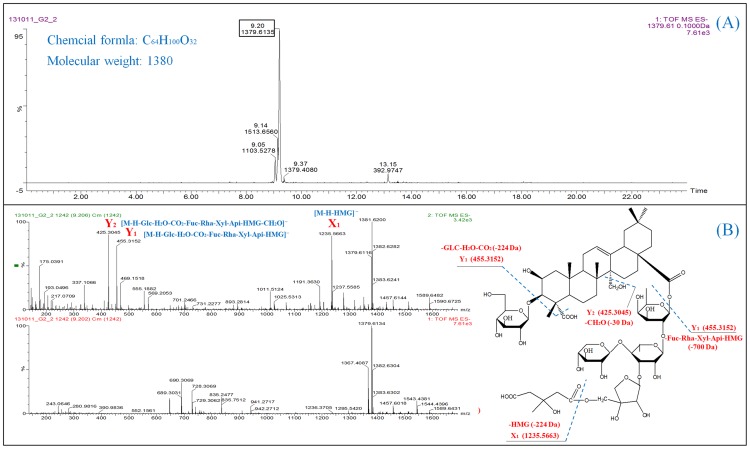
Extracted ion chromatogram (A) and MS/MS spectra (B) of Onjisaponin TG. Glc = β-D-glucopyranosyl; Fuc = β-D-fucopyranosyl; Xyl = β-D-xylopyranosyl; HMG = D 3-hydroxy-3-methyl-5-pentanoic acid ester.

The UPLC system provides a rapid, effective, and convenient method to analyze the chemical constituent's variance between different tissues in *P. tenuifolia*, whereas Q-TOF MS provides the accurate mass measurement. Figure 1 shows definite differences among the four samples from a visual examination of MS total ion current (TIC) chromatograms. A total of 25 compounds, including 4 xanthone C-glycosides, 6 sucrose esters, 8 oligosaccharide multi-esters, and 7 triterpene saponins, were identified. The details of identified compounds are summarized in Table 1. Figure 2 shows the typical MS/MS spectrum of Onjisaponin TG. The observation of [M-H-Glc-H_2_O-CO_2_-Fuc-Rha-Xyl-Api-HMG-CH_2_O]^−^ at m/z 455.3153 (Y_1_) and [M-H-Glc-H_2_O-CO_2_-Fuc-Rha-Xyl-Api-HMG]^−^ (Y_2_) at m/z 425.3045 in the MS/MS spectrum can be considered as the diagnostic ions for this type of triterpene saponins, and the fragment ions at m/z 1235.5663, corresponding to the losses of 144 Da proved the occurrence of 3-hydroxy-3-methyl-5-pentanoic acid ester (X_1_).

**Table 1:** Compounds identified in *P. Tenuifolia* by UPLC/Q-TOF MS.

	Formula	Identification	R_t_ (min)	Measured (m/z)	Calculated (m/z)	Maior fragment ions (m/z)	Reference(s)
**Sucrose esters**	C_22_H_30_H_14_	Sibiricoses A5	2.34	517.1551	517.1563	193.0496, 175.0396, 160.0160.	[67]
	C_23_H_32_O_15_	Sibiricoses A1	2.6	547.1661	547.1668	223.0630, 205.0529, 190.0264.	[67]
	C_30_H_36_O_17_	Tenuifoliside B	4.54	667.1863	667.188	461.1268, 281.0678, 205.0495, 190.0262, 137.0234.	[40]
	C_34_H_42_O_19_	3,6′-Disinapoyl sucrose	5.62	1507.4446	1507.4568	753.2266, 547.1672, 529.1566, 367.1044, 223.0619, 205.1518.	[67]
	C_31_H_38_O_17_	Tenuifoliside A	6.4	681.202	681.2036	443.1170, 281.0660, 223.0591, 179.0343, 137.0235.	[66]
	C_35_H_44_O_19_	Tenuifoliside C	7.45	767.2397	767.2404	529.1567, 367.1026, 237.0766, 223.0608, 190.0264.	[40]
**Xanthone and 4-xanthone C-glycosides**	C_24_H_26_O_14_	Sibiricaxanthone A	3.68	537.1239	537.125	405.0819, 387.0776, 315.0521, 297.0399, 285.0413, 267.0292, 243.0267.	[67]
	C_19_H_18_O_10_	Lancerin	3.6	405.0818	405.0822	315.0511, 285.0403, 257.0445.	[67]
	C_25_H_28_O_15_	Polygalaxanthone III	4.08	567.1347	567.1355	435.0929, 345.0636, 327.0503, 315.0511, 297.0372, 272.0313.	[67]
	C_20_H_20_O_11_	7-O-Methylmangiferin	3.97	435.0921	435.0933	345.0633, 315.0491, 272.0305.	[5]
**Oligosaccharide multiesters**	C_67_H_84_O_38_	Tenuifoliose L	7.55	1495.4456	1495.4568	1307.3792,1203.3612, 1161.3496, 1039.3143, 795.2366, 145.0289.	[41]
	C_55_H_68_O_31_	Tenuifoliose S	7.43	1223.3657	1223.3672	1101.3319, 955.2926, 631.1874, 145.0289.	[8]
	C_57_H_70_O_32_	Tenuifoliose K	7.63	1265.3757	1265.3777	1119.3420, 997.3044, 145.0291	[41]
	C_59_H_74_O_34_	Tenuifoliose P	8.13	1325.3955	1325.3989	1119.3442, 997.3010, 307.0816, 145.0296.	[41]
	C_58_H_72_O_33_	Tenuifoliose C	7.88	1295.3853	1295.3883	1119.3381, 997.3019, 851.2676, 175.0390, 145.0289.	[42]
	C_56_H_70_O_32_	Tenuifoliose T	7.76	1253.3757	1253.3777	1131.3373, 985.2862, 823.2463, 481.1313, 193.0488, 145.0289.	[8]
	C_60_H_74_O_34_	Tenuifoliose B	8.42	1337.3951	1337.3989	1161.3494, 1119.3401, 1101.3303, 1039.3107, 1021.2996, 175.0395.	[42]
	C_62_H_76_O_35_	Tenuifoliose A	9.37	1379.4059	1379.4094	1161.3502, 1143.3397, 1039.3156.	[42]
**Triterpenoid saponins**	C_53_H_84_O_24_	Polygalasaponin XXVIII	9.08	1103.5265	1103.528	455.3151, 425.3055.	[7]
	C_64_H_100_O_32_	Onjisaponin TG	9.2	1379.6062	1379.6125	1235.5663, 455.3152, 425.3045.	[8]
	C_82_H_122_O_41_	Onjisaponin V	12.4	1761.7027	1761.7389	1617.6771, 425.3066.	[11]
	C_86_H_128_O_43_	Onjisaponin L	12.03	1847.7335	1847.7757	1703.7048, 425.3055.	[7]
	C_75_H_112_O_35_	Senegin III	12.3	1571.6747	1571.6911	567.1912, 425.3048.	[7]
	C_81_H_120_O_40_	Onjisaponin W	12.81	1731.6967	1731.7283	1587.6743, 425.3052.	[11]
	C_65_H_96_O_28_	Onjisaponin TH	12.95	1323.5986	1323.6015	455.3159, 425.3062.	[8]

### Multivariate statistical analyses

**Figure 3:**
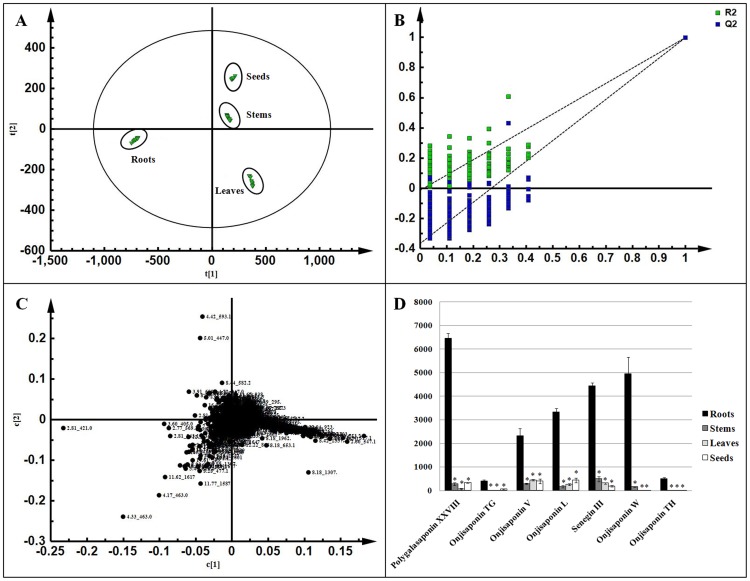
PCA scores plots derived from UPLC/Q-TOF MS data for extracts obtained from four different tissues (A); Permutation test with 200 permutations of PLS-DA model (B); PLS-DA loadings plot obtained from the metabolic profiles of four different tissues (C); Peak area intensity of triterpenoid saponins differences between the four tissue groups (D). ^*^ The stems, leaves, and seeds group compared with the roots group, *p*<0.05.

The normalized data sets from each sample column contained 1826 variables. The resulting integral data were imported in SIMCA-P for multivariate analysis. PCA was used first to detect any inherent trends within the data as an unbiased statistical method. PCA score plots showed that the groups of roots, stems, leaves, and seeds were clearly clustered into four groups (Figure 3A). To further study the variance between the different tissues and to determine potential biomarkers, supervised PLS-DA was subsequently used. The PLS-DA model was also validated by a permutation test with 200 permutations (Figure 3B) (R2 X = 0,929, Q2 = 0.925). The results indicated that the PLS-DA pattern combined with the loading plot was suitable to find out potential biomarkers.

### Potential biomarkers

Based on the normalized data of UPLC/Q-TOF MS separation, the loading plots (Figure 3C) were constructed for PLS-DA. The variables with VIP value >1.000 were marked as potential biomarkers. As shown in Table 2, a total of 22 marker compounds accountable for the different metabolite profiles of the roots, stems, leaves and seeds were observed. Sibiricoses A1, Sibiricoses A5, and Lancerin were the compounds particularly responsible for these variations.

**Table 2:** VIP values of PCA and peak area intensity of tissue metabolites measured by UPLC/Q-TOF MS.

No.	metabolite	Rt (min)_ m/z	Area (Mean ±SD)				VIP values	The highest group
			Roots	Stems	Leaves	Seeds		
**1**	Sibiricoses A1	2.60_547.1661	11368.28±260.96	674.93±37.98	571.80±19.43	743.56±44.29	4.325	Roots
**2**	Sibiricoses A5	2.34_517.1551	10554.63±292.87	473.91±25.15	446.51±13.46	889.27±50.27	4.202	Roots
**3**	Lancerin	3.60_405.0818	51.35±4.90	2032.58±85.61	4939.11±155.07	4348.47±186.85	4.041	Leaves
**4**	Polygalaxanthone III	4.08_567.1347	8197.06±2483.86	0.00±0.00	18.22±27.33	122.05±8.16	3.708	Roots
**5**	Tenuifoliose B	8.42_1337.3951	6170.21±170.41	381.67±42.20	585.98±28.76	370.39±52.23	3.271	Roots
**6**	Polygalasaponin XXVIII	9.08_1103.5265	6452.00±218.40	278.77±54.79	67.75±22.65	333.46±21.72	3.246	Roots
**7**	Sibiricaxanthone A	3.68_537.1239	5599.68±146.72	125.49±14.80	134.68±8.27	314.90±19.71	3.104	Roots
**8**	Onjisaponin W	12.81_1731.6967	4954.05±703.37	157.30±18.62	2.59±2.24	6.29±5.43	2.873	Roots
**9**	Tenuifoliside C	7.45_767.2397	4687.76±164.78	164.09±21.27	7.85±5.70	6.93±5.47	2.805	Roots
**10**	Senegin III	12.30_1571.6747	4451.49±120.50	494.98±109.48	313.29±30.11	189.03±38.69	2.652	Roots
**11**	Onjisaponin L	12.03_1847.7335	3346.83±134.03	169.47±43.40	256.64±34.85	432.41±97.86	2.402	Roots
**12**	3,6′-Disinapoyl sucrose	5.62_1507.4446	3316.47±152.63	146.15±15.14	0.57±0.25	3.16±2.29	2.343	Roots
**13**	Tenuifoliose K	7.63_1265.3757	2757.39±127.43	123.26±15.54	366.93±23.90	314.86±27.28	2.279	Roots
**14**	Tenuifoliose A	9.37_1379.4035	2722.79±80.79	142.62±41.57	6.72±5.42	7.22±5.49	2.113	Roots
**15**	Onjisaponin V	12.40_1761.7027	2333.99±296.19	286.96±32.90	443.00±30.61	400.14±94.15	1.972	Roots
**16**	Tenuifoliose S	7.43_1223.3657	1889.47±88.27	10.45±7.38	0.00±0.00	1.28±0.84	1.820	Roots
**17**	Tenuifoliose C	7.88_1295.3853	1919.92±70.24	65.49±25.60	36.86±9.06	54.28±9.69	1.799	Roots
**18**	Tenuifoliside B	4.54_667.1863	1603.98±68.85	7.91±15.77	0.00±0.00	0.00±0.00	1.678	Roots
**19**	Tenuifoliside A	6.40_681.2020	1819.80±686.52	282.38±91.52	10.59±4.06	12.06±4.38	1.609	Roots
**20**	Tenuifoliose L	7.55_1495.4456	1373.31±62.15	50.50±27.42	9.48±8.51	197.45±17.88	1.522	Roots
**21**	Tenuifoliose T	7.76_1253.3757	991.62±48.50	10.13±6.88	0.66±0.44	0.83±0.65	1.313	Roots
**22**	7-O-Methylmangiferin	3.97_435.0921	418.71±16.63	1082.99±121.96	1328.69±50.78	1139.79±70.92	1.091	Leaves

The main objective of this experiment is to focus on the changes of triterpenoid saponins between the different tissues. Five of the seven triterpenoid saponins had important functions in the intergroup differences. Furthermore, the peak area intensity of all seven triterpenoid saponins was analyzed (Figure 3D). In this experiment, the peak area intensity of most marker compounds were higher in the roots (*p*<0.05), whereas Lancerin and 7-O-Methylmangiferin were higher in the stems, leaves, and seeds (Table 2).

### Screening of reference genes of *P. tenuifolia*

**Figure 4:**
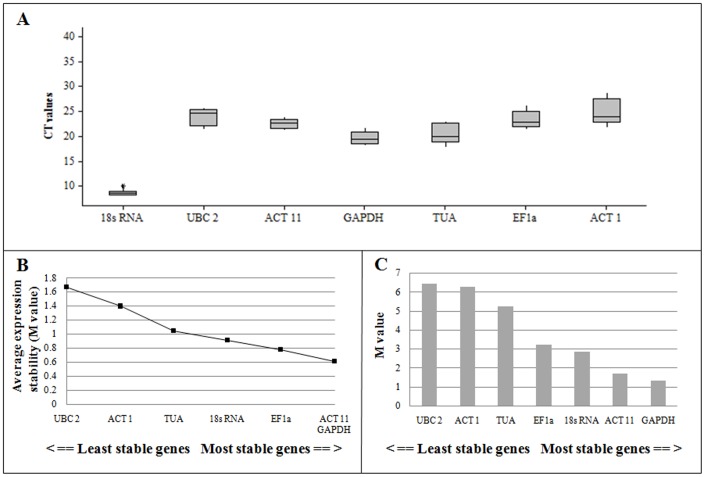
Expression levels of candidate reference genes in the different tissues by qRT-PCR (A); Average expression stability values (M) of the candidate reference genes calculated by geNorm (B); Gene expression stability and ranking of reference genes as calculated by NormFinder (C).

Seven candidate traditional reference genes including 18s ribosomal RNA (18s RNA), crystallinum ubiquitin-conjugating enzyme (UBC 2), actin 11 (ACT 11), glyceraldehyde-3-phosphate dehydrogenase (GAPDH), alpha tubulin (TUA), elongation factor 1-alpha (EF1α), and actin 1 (ACT 1) were selected (Table 3). Based on Figure 4A, the expression levels of the seven reference genes in different tissues varied extensively with Ct values ranging from 8 to 30 cycles. Most abundantly transcribed was 18s RNA with Ct value of less than 10 cycles. The rest of the genes were moderately expressed mRNAs with Ct values between 20 and 27 cycles.

**Table 3:** Primer sequences for qRT-PCR.

Gene	Annotation	Sequences		Product size (bp)
		Forward	Reverse	
**ACT 11**	Actin 11 Mrna	TTGGAAGGTGCTGAGGGAA	TGCTTAGTGGCGGAACGAC	167
**18 s RNA**	18S ribosomal RNA	CCTGTTATTGCCTCAAACTTCC	GTGGAGCGATTTGTCTGGTT	140
**GAPDH**	Glyceraldehyde-3-phosphate dehydrogenase	ACAGCAACGTGCTTCTCACC	CCCTTCATCACCACCGACTA	128
**TUA**	Alpha tubulin mRNA	TTCTCCTTCCTCCATACCCTC	ATCAACTACCAGCCTCCCACT	189
**EF1α**	Elongation factor 1-alpha mRNA	CAAAGGCACGGTATGATGAAAT	GTGGGACCCTTGTACCAGTCAA	157
**UBC 2**	Crystallinum ubiquitin-conjugating enzyme	CTCAACAATCTCCCGTACCCTT	GTCATTACTCTGCGACCCAAAC	109
**ACT 1**	ACT1 mRNA	TGTGATGGTTGGTATGGGACA	AGTTGCTAACAATTCCGTGCT	110
**MK**	Mevalonate kinase	CGGTTGTTCATGGATCTACTGC	TCAAGTCCCATATCCTTCAGC	128
**PMK**	Phosphomevalonate kinase	GTAGCCCTCGTGCTTCAATCT	TTTGCTGTAGCTGCTGCCTTT	145
**HDR**	4-hydroxy-3-methylbut-2-enyl diphosphate reductase	TTCCATCCACCAACTACCAGC	ATGCTACCCAAGAGCGACAAG	81
**HDS**	(E)-4-hydroxy-3-methylbut-2-enyl-diphosphate synthase	CAGGCATATCGTTTGCTTGTAG	CTTCATTCTCCCATCCTCACC	105
**FPS**	Farnesyl diphosphate synthase	CCATACAAGGCAAATCGTCAT	GCAATGCGCTACTCACTCCTC	166
**SQS**	Squalene synthase	TTCTATGCTTCACAAGGTCTCACG	ACATCTGTAGGGACGCTTGTATCA	144
**SQE**	Squalene monooxygenase	TATGAAGCGACCATTGTGAAAG	GCTCAACGGATCTATGGGTAT	120
**CAS**	Cycloartenol synthase	CTAATGCCCGGACTTGTAATCA	CCAAACATGGTGCTGTGACCT	158
**β-AS**	Beta-amyrin synthase	TACTGTATTCCCAGAAGAGCATC	GAATCCACTTTCTTGCTCTAACA	202
**CYP88D6**	Cytochrome P45088D6	CAGGAACCCAAGATTGACAGC	GCCATAGACTCTTTGGAGCAA	138
**CYP716B1**	Cytochrome P450716B1	ACTTGCTGGAACTGAGTGGTGA	CCATCTGTCCGATGTACTGCTT	99
**CYP72A1**	Cytochrome P45072A1	TTGCCTTGTAGACCCTGTTGT	AGCACTGTCGCATTGTCCTTA	125
**UGT 74B1**	UDP-glucosyl transferase 74B1	ATGAAGCAGGCTATGAACAAGC	CACATCAAGAGCCCAAGGAAAA	164
**UGT73B2**	UDP-glucosyl transferase 73B2	TCTGGGACTCCCTGATAACCT	AGTTCCGATTCAGAGCCTTTT	133
**UGT73C6**	UDP-glucosyl transferase 73C6	TTTGCTTTGGGAGTTGGTGTC	CCGTTTCATTATTGGCGTCTT	123

The geNorm software was used to analyze the gene expression stabilities of the seven reference genes. This software calculates the average expression stability value (M) based on the average pair-wise variation between all genes tested [43]. The gene with the lowest M value illustrates the most stable expression, whereas the highest M value indicates least stable expression. Except for UBC2, all genes had M value below the geNorm threshold of 1.5, whereas ACT 11 and GAPDH had the lowest M value (0.613) (Figure 4B). Therefore, ACT 11 and GAPDH had the most stable expression and UBC2 had the least stable expression.

The NormFinder software, whose criteria is the same with geNorm, was used to confirm results obtained by the geNorm program. Similar with the geNorm method, the gene with the lowest M value has the most stable expression, and the gene with the highest M value has the least stable expression. Results of NormFinder were showed in Figure 4C, GAPDH and ACT 11 had the most stable expression in different tissues. Stable genes identified in geNorm were confirmed by NormFinder, and GAPDH was chosen as the reference gene in the different tissues of *P. tenuifolia*.

### qRT-PCR analysis for genes involved in triterpenoid saponin biosynthesis pathways

**Figure 5:**
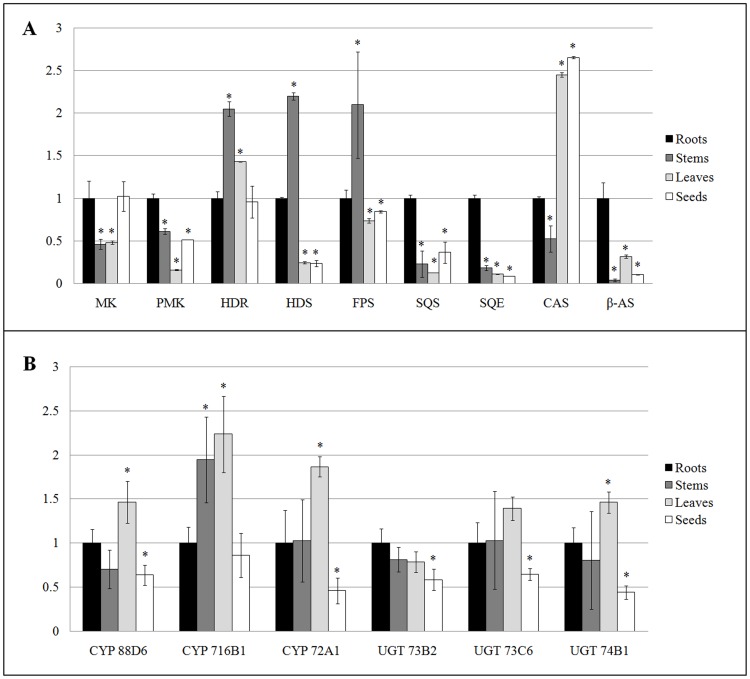
Expression pattern of genes involved in triterpenoid saponin backbone biosynthesis pathway in the different tissues of *P. Tenuifolia* by qRT-PCR (A); QRT-PCR analysis of CYP450s and UGTs in the different tissues of P. Tenuifolia (B). ^*^ The stems, leaves, and seeds group compared with the roots group, *p*<0.05.

To explore the mechanism of the triterpenoid saponin changes in the different tissues and understand its biosynthesis pathway in *P. tenuifolia*, the 15 genes (Table 3) putatively expressed in triterpenoid saponin biosynthesis pathway were analyzed by qRT-PCR. As shown in Figure 5A, among the nine genes involved in triterpenoid saponin backbone biosynthesis, phosphomevalonate kinase (PMK), SQS, SQE, and β-AS genes in the roots were significantly overexpressed with 2 to 13-fold higher expression than the other tissues (*p*<0.05). The expression level of 4-hydroxy-3-methylbut-2-enyl diphosphate reductase (HDR), (E)-4-hydroxy-3-methylbut-2-enyl-diphosphate synthase (HDS), and farnesyl diphosphate synthase (FPS) in the stems were significantly higher compared with that in the roots (*p*<0.05). Moreover, cycloartenol synthase (CAS) genes exhibited the highest expression in the seeds.

Furthermore, the correlation coefficients between the peak area intensity of triterpenoid saponins and expression levels were computed by SPSS. The expression levels of SQS, SQE, and β-AS showed high correlation with the peak area intensity of triterpenoid saponins in different tissues (Table 4). The transformation of trans,trans-farnesyl diphosphate (FPP) to (S)-2,3-oxidosqualene requires the function of SQS and SQE [35]. The expression of SQE in the roots was significantly over expressed with an average of six fold compared with the other tissues (*p*<0.05). The next important step in the pathway is through β-AS and CAS, which catalyzes 2,3-oxidosqualene to form triterpenoid saponins and sterol, respectively [36]. The expression level of CAS was higher in the leaves and seeds compared with the root (*p*<0.05), whereas the β-AS expression in the root was higher (*p*<0.05) and overexpressed with an average of 13 fold compared with the stems, leaves, and seeds.

**Table 4:** The correlation coefficient between the peak area intensity of triterpenoid saponins and genes expression.

	Peak area intensity	MK	PMK	HDR	HDS	FPS	SQS	SQE	CAS	β-AS	CYP88D6	CYP716B1	CYP72A1	UGT73B2	UGT73C6	UGT74B1
Peak area intensity	1.00															
MK	0.31	1.00														
PMK	0.55	0.54	1.00													
HDR	−0.31	−0.78	−0.23	1.00												
HDS	0.07	−0.39	0.40	0.76	1.00											
FPS	−0.06	−0.44	0.20	0.73	0.88	1.00										
SQS	0.72	0.56	0.87	−0.45	0.09	−0.09	1.00									
SQE	0.69	0.45	0.85	−0.31	0.16	−0.06	0.94	1.00								
CAS	−0.31	0.22	−0.63	−0.58	−0.93	−0.74	−0.40	−0.50	1.00							
β-AS	0.69	0.43	0.66	−0.44	−0.11	−0.30	0.88	0.93	−0.25	1.00						
CYP88D6	−0.05	−0.24	−0.35	0.00	−0.40	−0.49	−0.06	0.10	0.27	0.33	1.00					
CYP716B1	−0.35	−0.77	−0.49	0.70	0.29	0.23	−0.55	−0.35	−0.17	−0.30	0.48	1.00				
CYP72A1	−0.20	−0.48	−0.40	0.32	−0.10	−0.31	−0.26	−0.08	0.05	0.09	0.73	0.65	1.00			
UGT73B2	0.51	−0.15	0.46	0.16	0.37	0.19	0.48	0.60	−0.56	0.59	0.17	0.15	0.23	1.00		
UGT73C6	−0.03	−0.43	−0.26	0.29	0.02	−0.02	−0.21	0.02	−0.08	0.07	0.49	0.62	0.50	0.19	1.00	
UGT74B1	0.11	−0.33	−0.24	0.20	−0.12	−0.27	−0.01	0.18	0.00	0.35	0.80	0.43	0.80	0.30	0.46	1.00

The correlation between the gene expression level and peak area of compound also represents an approach to determine the possible CYP 450 and UGT genes involved in the triterpenoid saponin biosynthesis. However, no apparent regularity in the expression of CYP88D6, CYP716B1, CYP72A1, UGT74B1, UGT73B2, and UGT73C6 (Figure 5B) was observed. Results demonstrated that UGT73B2 mRNA levels were highest in the roots and the expression levels of CYP88D6, CYP716B1, CYP72A1, UGT74B1, and UGT73C6 were highest in leaves, respectively. However, the expression of these genes was significantly different from SQS, SQE, and β-AS. In addition, the correlation coefficient between the gene expression level and peak area intensity of triterpenoid saponins were less than 0.50 (Table 4).

## Discussion

**Figure 6:**
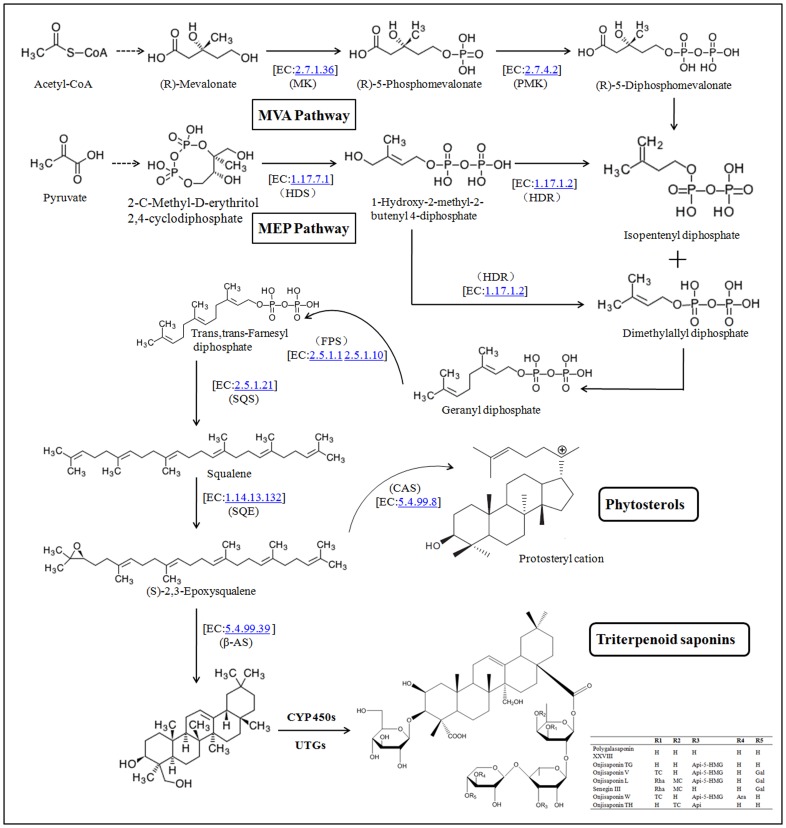
Putative triterpenoid saponin biosynthesis pathway in *P. tenuifolia.*

The recent advances in metabolomics have provided new insights in the meaningful biological changes of numerous metabolites using liquid chromatography-mass spectrometry (LC-MS), gas chromatography-mass spectrometry (GC-MS), and nuclear magnetic resonance (NMR) technologies. In this study, LC-MS metabolomic profiles have clearly demonstrated the changes of secondary metabolites particularly the triterpenoid saponins in the different tissues of *P. tenuifolia*. Furthermore, the nine enzyme genes involved in the triterpenoid saponin backbone biosynthesis and six enzyme genes involved in the oxygenation and glycosylation of cyclization product (Figure 6) were analyzed. The understanding of the biological significance of the alteration of these triterpenoid saponins and genes may provide evidence for further discovery of enzyme gene with key role in *P. tenuifolia*.

### UPLC/Q-TOF MS analysis and accumulation of triterpenoid saponins

Considering the complexity of the plant metabolite composition, plant metabolomic analyses have many challenges. Compared with GC-MS and NMR technologies, HPLC technology has an advantage on the analysis of plant secondary metabolites. Currently, HPLC ultraviolet detector (HPLC-UV), HPLC evaportive light scattering detector (HPLC-ELSD), HPLC photodiode array detector (HPLC-PDA), and UPLC/Q-TOF MS methods have been developed for the qualitative and/or quantitative analysis of plant secondary metabolites. Among these analytical methods, the UPLC coupled with Q-TOF MS has become a powerful tool for metabolite profiling and identifying the complicated components in herbal medicine [44]–[45]. In previous studies, positive and negative ion modes were investigated. The result were that the detection of saponins is less difficult using negative ion mode and the confirmation of molecular ions or quasimolecular ions for the identification of each peak [42] is also easier. Thus, mass data monitored with negative ion mode were utilized for component characterization.

Triterpenoid saponins, as a category of secondary metabolites, indirectly participate in plant growth and development. To protect the plant from herbivore and to be part of the plants' defense systems (anti-microbial, anti-fungicidal, and insecticidal) are the most important functions of triterpenoid saponins in plants [46]. The triterpenoid saponin accumulations are influenced by several environmental factors, such as seasonal variation, nutrient, light irradiation, or its combined effects. The distribution of triterpenoid saponins also demonstrates seasonal fluctuations or greatly varies in the individual plant organs or tissues. For instance, Lin et al. [47] found that peak area intensity of saponins in the tubers of *Dioscorea pseudojaponica* is the maximum protection provider for this reproductive organ. Saponins, whose levels were increased in the early stages of berry development of *Phytolacca dodecandra*, also prevent fruit loss and ensure seed maturation [48].

In this study, result of metabolomic analysis showed a clear separation in the PCA space of the different tissues of *P. tenuifolia* analyzed by UPLC/Q-TOF MS. Considerable information in biomarkers were obtained through accurate mass measurement and MS/MS analysis. The specific accumulation of triterpenoid saponins in the root of *P. tenuifolia* indicated that it might counteract soil-borne fungi, which is similar with the accumulation of saponins in the root of *Avenae strigosa* [49]. Moreover, the different peak area intensity and distribution of triterpenoid saponins in different tissues provide an excellent model to research its biosynthesis pathway and screen enzyme gene with key role in its biosynthesis.

### Selection of reference genes in *P. tenuifolia*

The qRT-PCR technique is one of the most common methods to quantify gene expression levels in order to understand biological processes. However, a reliable method to normalize its expression is necessary. Thus, an appropriate internal reference gene is required for reliable quantification of gene transcripts [50]. We examined the expression levels of seven reference genes obtained from transcriptome data of *P. tenuifolia* in different tissues. We also analyzed their expression using geNorm and NormFinder. Based on these softwares, data showed that top two positions of the reference genes for the different tissue samples were similar. Because GAPDH is a commonly used reference gene to normalize the gene expression data in qRT-PCR assay [51], it was also chosen as the reference gene in the different tissues of *P. tenuifolia*.

### Biosynthesis of triterpenoid saponin backbone

Current ideas consider that the early stage of triterpenoid saponin biosynthesis in plants starts from the cytosolic MVA and plastidial MEP pathway [17]. The MVA pathway converted acetyl-CoA to isopentenyl diphosphate (IPP), whereas MEP pathway started from pyruvate to IPP and then to dimethylallyl diphosphate (DMAPP) (Figure 6). However, less progress has been made toward determining the precise source of isoprene units, and clarifying what the pathway is important. In this study, we determined the gene expression of MK and PMK of MVA pathway, and HDR and HDS of MEP pathway. Results showed that gene expression levels of MK and PMK were generally high in the roots, whereas HDR and HDS were higher in the stems. The correlation coefficient showed that gene expression of MK and PMK had higher correlation with the peak area intensity of triterpenoid saponins than HDR and HDS (Table 4). Result suggested that MVA pathway has more important functions in the triterpenoid saponin biosynthesis of *P. tenuifolia*.

Geranyl diphosphate (GPP) is obtained through the condensation of IPP and DMAPP, and FPP, which is the common precursor of the vast array of sesquiterpenes, resulted from the addition of a second IPP unit. Two trans,trans-FPP molecules are facilitated by SQS to produce squalene. Squalene is oxidized by SQE to (S)-2,3-oxidosqualene, which is the first step leading to cyclizations and common precursor of phytosterols and triterpenoid saponins [51]. Moreover, β-AS facilitates (S)-2,3-oxidosqualene backbone into a chair–chair–chair conformation in contrast with the chair–boat–chair conformation during CAS catalysis. Based on the experimental results, the gene expression levels of SQS, SQE, and β-AS in the root were significantly higher than in the other tissues. With regard to the total peak area intensity of triterpenoid saponins, the correlation coefficients between the above three gene expression levels and peak area intensity were higher than the others. This result indicated that SQS, SQE, and β-AS have important functions in the triterpenoid saponin biosynthesis pathway of *P. tenuifolia*. Mangas et al. [52] suggested that SQS and β-AS have regulatory function by comparing triterpenoid saponin peak area intensity with the expression level of some related genes in the cauli of *Centella asiatica*. Han et al. [53] demonstrated that squalene enhances the accumulation of SQE with SQS, dammarenediol II synthase (DDS), β-AS, and cycloartenol synthase (PNX) by RT-PCR analysis in *Panax ginseng*. Considering the present experiment result, the overexpression of SQS, SQE, and β-AS genes may be increasing the triterpenoid saponin production in *P. tenuifolia*.

### Design of the cyclization product - oxygenation and glycosylation

As compared with onjisaponin backbone biosynthesis, less information about the late stages (from sapogenin to various saponin) of triterpenoid saponin biosynthesis is available. Most common sapogenin modifications are oxygenation catalyzed by CYP450s protein families and glycosylation catalyzed by UGTs at various positions of the backbone [54]–[55]. Prior to this study, few publications report the identification of CYP450s and the involvement of UGTs in the biosynthesis of triterpenoid saponins (Table 5), and the identified genes from *P. tenuifolia* have not yet been accounted.

**Table 5:** CYP450s and UGTs involved in triterpenoid saponin biosynthesis.

Name	Species	Gene function	Reference(s)
**CYP93E1**	*Glycine max* (L.) Merr	Catalyze the hydroxylation of β-amyrin and its 22-hydroxyderivate sophoradiol at position C24	Shibuya et al. (2006) [56]
**CYP51H10**	*Avena strigosa* Schreb.	Not biochemically characterized	Qi et al. (2006) [57]
**CYP88D6**	*Glycyrrhiza uralensis* Fisch.	Catalyze C11 oxygenation of β-amyrin	Seki et al. (2008) [58]
**CYP93E3**	*Glycyrrhiza uralensi* Fisch.	Catalyze 24-hydroxylation of β-amyrin	Seki et al. (2008) [58]
**CYP93E2**	*Medicago truncatula* L.	Not biochemically characterized	Naoumkina et al. (2010) [59]
**CYP716A12**	*Medicago truncatula* L.	Catalyze C28 oxygenation of β-amyrin	Carell et al. (2011) [60]
**CYP72A154**	*Glycyrrhiza uralensis* Fisch.	Catalyze C30 oxygenation of β-amyrin	Seki et al. (2011) [61]
**UGT73P2**	*Glycine max* (L.) Merr	Extend the C3 saccharide moiety of the monoglycosylated oleanane aglycone soyasapogenol B with a galactosyl	Shibuya et al. (2010) [62]
**UGT91H4**	*Glycine max* (L.) Merr	Catalyze the third glycosylation step in soyasaponin I biosynthesis	Shibuya et al. (2010) [62]
**UGT73F3**	*Medicago truncatula* L.	Catalyze glucosylation of hederagenin and other oleanane sapogenins at the C28-carboxy group	Naoumkina et al. (2010) [59]
**UGT74M1**	*Saponaria vaccaria*	Catalyze linkage of glucosyl-moieties to the C28-carboxy group of preferably oleanane type sapogenins through an ester bond	Meesapyodsuk et al. (2007) [63]
**UGT71G1**	*Medicago truncatula* L.	Position specificity not elucidated	Achnine et al. (2005) [64]
**UGT73K1**	*Medicago truncatula* L.	Position specificity not elucidated	Achnine et al. (2005) [64]

In our previous research, a total of 466 and 157 gene reads are annotated as CYP450s and UGTs in the transcriptome of *P. tenuifolia* (data not shown), respectively. Based on the transcriptome data, six candidate genes, including CYP450 and UGT, which are in the same family of the identified P450s and UGTs involved in the biosynthesis of triterpenoid saponins, are selected for qRT-PCR analysis in different tissues. Seki et al [61] have reported that CYP88D6 expression is detected in the roots and stolons by RT-PCR, but no amplification is observed in the leaves or stems of *Glycyrrhiza* (licorice) plants. However, in the current study, the CYP88D6 expression in the leaves or stems was observed and did not show much difference among the different tissues. Carelli et al [60] have identified that CYP716A12 is involved in saponin synthesis of *Medicago truncatula* using a combined genetic and biochemical approach. However, in vitro enzymatic activity assays indicate that CYP716A12 catalyzes the oxidation of β-amyrin and erythrodiol at the C-28 position, generating oleanolic acid. Nevertheless, the gene expression of CYP716B1, from the same family of CYP716A12, was detected in the roots, stems, leaves, and seeds of *P. tenuifolia* and showed no correlation with triterpenoid saponin peak area intensity in the present research. Moreover, Seki et al [61] demonstrated that CYP72A154 expressed in an engineered yeast strain, which endogenously produced 11-oxo-b-amyrin (a possible biosynthetic intermediate between b-amyrin and glycyrrhizin) and catalyzed 3 sequential oxidation steps at C-30 of 11-oxo-b-amyrin, may supply in situ to produce glycyrrhetinic acid. This research also showed that CYP72A154 was expressed in the roots, stolons, and stems, whereas no transcripts were observed in the leaves of *Glycyrrhiza* plants [61]. However, in this study, the transcripts of CYP72A1 were not only detected in the roots, seeds, and stems, but also in the leaves of *P. tenuifolia*.

Regarding CYP450s genes, some UGT genes have also been cloned and identified. Research showed that UGT74M1 was identified by screening UGT sequences among ESTs of *Saponaria vaccaria* and catalyzed linkage of glucosyl-moieties to the C28-carboxy group of preferably oleanane-type sapogenins through an ester bond [63]. Furthermore, UGT73F3 was identified in *Medicago truncatula* and catalyzed glucosylation of hederagenin and other oleanane sapogenins at the C28-carboxy group [59]. In addition, the possible involvement in the glycosylation of brassinosteroids of other characterized UGTs of this family such as UGT73C5, UGT73C6, UGT73C8, UGT73F1, and UGT73P1 have also been reported [65]. The majority of UGTs involved in glycosylation belong to the UGT73 and UGT74 families. In the current study, the gene expression of UGT74B1, UGT73B2, and UGT73C6 did not show much correlation with the peak area intensity of triterpenoid saponins, which is similar with three CYP450 genes in *P. tenuifolia*.

## Conclusions

In summary, significant differences in all 7 triterpenoid saponins among the different tissues were found. GAPDH was chosen as the reference gene in the different tissues of *P. tenuifolia*. Results from this study suggested that MVA pathway has more important functions and SQS, SQE, and β-AS are the enzyme genes with key role in the triterpenoid saponin biosynthesis of *P. tenuifolia*. This result was established through the correlation analysis between the triterpenoid saponin peak area intensity and expression levels of 15 relevant genes involved in the biosynthesis. The metabolomic analysis by UPLC/Q-TOF MS combined with the gene expression analysis by qRT-PCR technique provide useful information in studying gene discovery and an effective approach in understanding the mechanism of onjisaponin biosynthesis. Along with the technology advances and its broader availability, the digital gene expression profile (DEG)-based generation sequencing or DNA microarray-based gene expression cluster analysis may also facilitate gene discovery.

## Materials and Methods

### Materials and reagents

The fresh roots, stems, leaves, and seeds from the samples of *P. tenuifolia*, were collected in Xiongshan forest farm, mountain of Wulong, Bayi town, Changzhi county, Shanxi province (latitude: N35°57′59.98″; longitude: E113°01′36.58″). No specific permits were required from the forest farm to select samples. The forest farm is not privately-owned and the field studies did not involve protected species. Each sample was divided into two parts, in which one part was used for UPLC/Q-TOF MS analysis. The other parts were collected in RNase-free tubes and stored frozen in liquid nitrogen at −80°C.

Acetonitrile of UPLC grade were obtained from Fisher Scientific Co., Ltd. (Waltham, MA, USA). Formic acid and analytic-grade methanol were purchased from Fisher Scientific Co., Ltd. (Waltham, MA, USA). RNAiso Plus kit, PrimerScript RT master mix perfect real-time kit, and SYBR Premix Ex Taq II kit were purchased from TAKARA Co., Ltd. (Dalian, China). Primers were synthesized from Sangon Biotech (Shanghai, China).

### Sample preparation for UPLC/Q-TOF MS analysis

Roots, stems, leaves, and seeds of 9 individual *P. tenuifolia* plants were sequentially pulverized in a mortar with liquid nitrogen. A total of 1 g drug powder of each sample was extracted for 30 min by ultrasonication in 10 mL of methanol, and then the sample solution was filtered through a 0.22 µm filter membrane before use. A 2 µL aliquot was used for UPLC/Q-TOF MS analysis.

### UPLC/Q-TOF MS analysis

*P. tenuifolia* metabolite profiling was performed on a Waters ACQUITY UPLC I-Class/Xevo in line with a Waters Xevo G2 Q-TOF mass spectrometer (Waters Corporation, Milford, MA, USA). Samples were separated on a Zorbax Eclipse Plus Acquity UPLC BEH C 18 (1.7 µm particle size) 2.1 mm×50 mm. The column temperature was maintained at 40°C. The flow rate was 0.50 mL/min. The mobile phase consisted of acetonitrile (A) and water containing 0.1% (v/v) formic acid. The gradient was initiated with increasing to 5% A within 1 min, 5% A and then linearly increased to 12% A within 2 min, then 12% A increased to 20% A within 3 min, 20% A increased to 30% A within 4 min, and 30% A increased to 35% A within 0.5 min. Next, 35% A increased to 45% A within 3 min and 45% A was increased to 50% A within 1.5 min. Finally, 50% A increased to 95% A within 3 min and 95% A increased to 100% A within 1 min.

The Xevo G2-S Q-TOF mass spectrometer was run in negative mode. The data were collected for each test sample from 50 Da to 1500 Da. High purity nitrogen (N_2_) was used as nebulizing gas, and ultra-high pure helium (He) as collision gas. Source parameters were as follows: capillary voltage, 2.50 kV; sampling cone voltage, 40.0 V; source offset, 100 V; desolvation temperature, 500°C; cone gas flow, 50 L•h^−1^; and desolvation gas flow, 800 L•h^−1^. To ensure mass accuracy and reproducibility, leucine-enkephalin was used as the reference lock mass (m/z 554.2615) with the LockSpray interface.

### UPLC/Q-TOF MS data acquisition and analysis

Waters' metabolomics package, namely TransOmics, developed with Nonlinear Dynamics, can process large sample sets, automatically detect features, and perform a quantitative comparison with retention time (R_T_) and m/z data pairs as identifiers. The result of roots, stems, leaves and seeds samples were imported to SIMCA-P13.0 software package (version 13.0, Umetrics, Umeå, Sweden) for multivariate statistical analysis.

Principal component analysis (PCA) and partialleast-squares-discriminant analysis (PLS-DA) were run to separate between different tissue groups. Pareto scaling was used in all the models to avoid chemical noise. To evaluate the quality of the model, R2 and Q2 values were also calculated. R2 displays the variance explained in the model and indicates the goodness of fit. Q2 displays the variance in data predicted by the model and indicates predictability. Potential biomarkers were selected according to the loading plot and Variable Importance in the Project (VIP) values. Compound identification of metabolites was performed based on their retention times, exact mass information and fragment ions according to previous studies [5], [7], [8], [66], [67]. The peak area intensity analysis of potential biomarkers and triterpenoid saponins were also run to find the variance between different tissue groups.

#### Total RNA extraction and cDNA synthesis

Total RNA was extracted from the different tissues by using RNAiso Plus kit according to the manufacturer's protocol. RNA concentration and purity were estimated from the optical density at A_260_/A_280_ ratio using the NanoDrop 2000 Spectrophotometer (Thermo Fisher Scientific, Wilmington, DE). The integrity of RNA was evaluated by electrophoresis. cDNA was amplified from the total RNA (500 ng) by using a PrimerScript RT master mix perfect real-time kit.

### qRT-PCR analysis

qRT-PCR was performed using the ABI StepOne/StepOne Plus (Applied Biosystems, USA) with a final volume of 20 µL containing cDNA, SYBR Green PCR master mix, ROX and primers. All qRT-PCR reactions were carried out in technical and biological triplicate. The amplification procedure included three stages: a holding stage for 10 min at 95°C; a cycling stage with 40 cycles consisting of 15 s at 95°C for denaturation and 60 s at 60°C for primer annealing; and a melt-curve stage consisting of 15 s at 95°C, 1 min at 60°C and 15 s at 95°C [68].

Melt-curve analysis was performed to confirm amplicon specificity. All the primers for the 7 reference genes and 15 putative genes involved in triterpenoid saponin biosynthesis used for amplification were designed using Primer 5 software, using the following criteria: melting temperatures of 59°C to 61°C, primer lengths of 21 bp to 25 bp and amplicon lengths of 80 bp to 202 bp (Table 1). The gene used as an internal control was chosen by the mean threshold cycle (Ct) values. Data were then presented according to the 2^−ΔΔCT^ method [69]. Measures were obtained for each condition from cDNA, which had been synthesized from RNA extracted from three independent cultures, each in two biological replicates.

### Statistical analyses

To select the suitable reference gene, GeNORM (version 3.5) [31] and NormFinder (version 0.953) [32] were used to analyze the stability of gene expression. The 2^−ΔΔCT^ data obtained from qRT-PCR were expressed as mean ±SD and analyzed in SPSS 16.0 software (SPSS Inc., Chicago, IL). Correlation analysis between peak area intensity of triterpenoid saponins and gene expression levels were performed, and differences were scored as statistical significance at *p*<0.05 by independent sample *t*-test by SPSS 16.0.

## Supporting Information

Table S1 **Summary of the annotation sources for reference genes of** ***P. Tenuifolia*** **.**
(DOC)

Table S2 **Summary of the annotation sources for relevant genes in triterpenoid saponins backbone biosynthesis pathway of** ***P. Tenuifolia*** **.**
(DOC)

Table S3 **Summary of the annotation sources for CYP450s and UGTs genes of** ***P. Tenuifolia*** **.**
(DOC)
