# Supplementary material for: UPLC/Q-TOF MS-Based Metabolomics and qRT-PCR in Enzyme Gene Screening with Key Role in Triterpenoid Saponin Biosynthesis of Polygala tenuifolia
Source: PLoS One. 2014 Aug 22;9(8):e105765. doi: 10.1371/journal.pone.0105765 (PMC4141818; doi:10.1371/journal.pone.0105765)
Supplement: Table S1 — Summary of the annotation sources for reference genes of P. Tenuifolia . (DOC) [file pone.0105765.s001.doc]

*Table S1 Summary of the annotation sources for reference genes of P. tenuifolia.*

| Gene | Unigene | Length(bp) | Accession No. (NCBI Nt) | Annotation | source | E-valule |
| --- | --- | --- | --- | --- | --- | --- |
| 18s RNA | Unigene 11 | 2237 | gb|AY929358.1|  (NCBI Nt) | 18S ribosomal RNA gene, partial sequence | *Perrottetia ovata* | 0 |
| UBC 2 | Unigene 279 | 1088 | gb|HQ323247.1|  (NCBI Nt) | Ubiquitin-conjugating enzyme 2 | *Hevea brasiliensis* | 1.00E-136 |
| ACT 11 | Unigene 3694 | 1717 | gb|AY305732.1|  (NCBI Nt) | Actin (ACT11) mRNA, complete cds | *Gossypium hirsutum* | 0 |
| GAPDH | Unigene 7303 | 2023 | gb|JQ302964.1|  (NCBI Nt) | Glyceraldehyde-3-phosphate dehydrogenase complete cds | *Petunia hybrida* | 0 |
| TUA | Unigene 65 | 1652 | gb|FJ228477.1|  (NCBI Nt) | Alpha tubulin mRNA, complete cds | *Betula pendula* | 0 |
| EF1a | Unigene 326 | 1558 | gb|JN399225.1|  (NCBI Nt) | Elongation factor 1-alpha mRNA, complete cds | *Rosa multiflora* | 0 |
| ACT 1 | Unigene 2689 | 1723 | gb|FJ485727.1|  (NCBI Nt) | Actin (ACT1) mRNA | *Caragana korshinskii* | 0 |
