# Supplementary material for: UPLC/Q-TOF MS-Based Metabolomics and qRT-PCR in Enzyme Gene Screening with Key Role in Triterpenoid Saponin Biosynthesis of Polygala tenuifolia
Source: PLoS One. 2014 Aug 22;9(8):e105765. doi: 10.1371/journal.pone.0105765 (PMC4141818; doi:10.1371/journal.pone.0105765)
Supplement: Table S2 — Summary of the annotation sources for relevant genes in triterpenoid saponins backbone biosynthesis pathway of P. Tenuifolia . (DOC) [file pone.0105765.s002.doc]

*Table S2 Summary of the annotation sources for relevant genes in triterpenoid saponins backbone biosynthesis pathway of P. tenuifolia.*

| Genes | Unigene | Length (bp) | Accession No. | Annotation | Pathway | E-valule |
| --- | --- | --- | --- | --- | --- | --- |
| MK | Unigene 15119 | 1582 | rcu:RCOM_1431350  (KEGG No.) | K00869 mevalonate kinase [EC:2.7.1.36] | Terpenoid backbone biosynthesis [PATH:ko00900-Map:ko00900] | 1.00E-125 |
| PMK | Unigene 11179 | 1698 | rcu:RCOM_0699810  (KEGG No.) | K00938 phosphomevalonate kinase [EC:2.7.4.2] | Terpenoid backbone biosynthesis [PATH:ko00900-Map:ko00900] | 0 |
| HDR | Unigene 7211 | 2125 | pop:POPTR_557296  (KEGG No.) | K03527 4-hydroxy-3-methylbut-2-enyl diphosphate reductase (EC:1.17.1.2) | Terpenoid backbone biosynthesis [PATH:ko00900-Map:ko00900] | 0 |
| HDS | Unigene 9670 | 2636 | vvi:100257071  (KEGG No.) | K03526 (E)-4-hydroxy-3-methylbut-2-enyl-diphosphate synthase [EC:1.17.7.1] | Terpenoid backbone biosynthesis [PATH:ko00900-Map:ko00900] | 0 |
| FPS | Unigene 33526 | 1489 | vvi:100266842  (KEGG No.) | K13789 geranylgeranyl diphosphate synthase, type II [EC:2.5.1.1 2.5.1.10 2.5.1.29] | Terpenoid backbone biosynthesis [PATH:ko00900-Map:ko00900] | 1.00E-122 |
| SQE | Unigene 2806 | 3434 | vvi:100265235  (KEGG No.) | K00511 squalene monooxygenase [EC:1.14.99.7] [EC:1.14.13.132] | Steroid biosynthesis [PATH:ko00100-Map:ko00100] | 0 |
| CAS | Unigene 15078 | 2807 | vvi:100243389  (KEGG No.) | K01853 cycloartenol synthase [EC:5.4.99.8] | Steroid biosynthesis [PATH:ko00100-Map:ko00100] | 0 |

| Gene | Length  (bp) | Accession No. | Annotation | Pathway |
| --- | --- | --- | --- | --- |
| SQS | 1965 | DQ672339.1  (GenBank NO.) | Polygala tenuifolia squalene synthase (SQS) mRNA, complete cds [EC:2.5.1.21] | Steroid biosynthesis [PATH:ko00100-Map:ko00100] |
| β-AS | 2934 | EF107623.1  (GenBank NO.) | Polygala tenuifolia beta-amyrin synthase mRNA, complete cds [EC:5.4.99.39] | Steroid biosynthesis [PATH:ko00100-Map:ko00100] |
