# Supplementary material for: UPLC/Q-TOF MS-Based Metabolomics and qRT-PCR in Enzyme Gene Screening with Key Role in Triterpenoid Saponin Biosynthesis of Polygala tenuifolia
Source: PLoS One. 2014 Aug 22;9(8):e105765. doi: 10.1371/journal.pone.0105765 (PMC4141818; doi:10.1371/journal.pone.0105765)
Supplement: Table S3 — Summary of the annotation sources for CYP450s and UGTs genes of P. Tenuifolia . (DOC) [file pone.0105765.s003.doc]

*Table S3 Summary of the annotation sources for CYP 450s and UGTs genes of P. tenuifolia.*

| Gene | Unigene | Length  (bp) | Accession No. | Annotation | E-valule |
| --- | --- | --- | --- | --- | --- |
| CYP88D6 | Unigene  655 | 2293 | sp|B5BSX1|BAMO_GLYUR  (SWISSPROT No.) | Beta-amyrin 11-oxidase OS=Glycyrrhiza uralensis GN=CYP88D6 PE=1 SV=1 | 5.00E-83 |
| CYP716B1 | Unigene  26568 | 1828 | sp|Q50EK1|C16B1_PICSI  (SWISSPROT No.) | Cytochrome P450 716B1 OS=Picea sitchensis GN=CYP716B1 PE=2 SV=1 | 1.00E-104 |
| CYP72A1 | Unigene  1714 | 2272 | sp|Q05047|C72A1_CATRO  (SWISSPROT No.) | Secologanin synthase OS=Catharanthus roseus GN=CYP72A1 PE=2 SV=1 | 1.00E-106 |
| UGT74B1 | Unigene  16901 | 1631 | sp|O48676|U74B1_ARATH  (SWISSPROT No.) | UDP-glycosyltransferase 74B1 OS=Arabidopsis thaliana GN=UGT74B1 PE=1 SV=1 | 1.00E-126 |
| UGT73B2 | Unigene  28122 | 1862 | sp|Q94C57|U73B2_ARATH  (SWISSPROT No.) | UDP-glucosyl transferase 73B2 OS=Arabidopsis thaliana GN=UGT73B2 PE=1 SV=1 | 1.00E-116 |
| UGT73C6 | Unigene  3377 | 1815 | sp|Q9ZQ95|U73C6_ARATH  (SWISSPROT No.) | UDP-glycosyltransferase 73C6 OS=Arabidopsis thaliana GN=UGT73C6 PE=2 SV=1 | 3.00E-97 |
